# Prevalence of depressive symptoms and its associated factors among the aging population in Gamo zone, Southern Ethiopia: a community-based cross-sectional study

**DOI:** 10.3389/fpsyt.2024.1402622

**Published:** 2024-08-30

**Authors:** Bilcha Oumer, Rahel Abera, Asrat Beshah, Selamnesh Tesfaye, Tilhun Niguse, Bedria Mohammed, Awol Arega Yimer, Negussie Boti Sidamo

**Affiliations:** ^1^ Department of Midwifery, Arba Minch Health Science College, Arba Minch, Ethiopia; ^2^ Department of Public Health, Arba Minch Health Science College, Arba Minch, Ethiopia; ^3^ Department of Midwifery, College of Medicine and Health Sciences, Arba Minch University, Arba Minch, Ethiopia; ^4^ School of Public Health, College of Medicine and Health Sciences, Arba Minch University, Arba Minch, Ethiopia

**Keywords:** depressive symptoms, associated factors, aging population, community based cross-sectional, Gamo zone, Southern Ethiopia

## Abstract

**Background:**

Depression in the elderly is becoming a major public health problem worldwide. It is a major public health problem associated with increased morbidity, mortality, and healthcare costs in low- and middle-income countries, including Ethiopia. However, especially in developing countries, they usually go undetected and untreated. There is little evidence of depressive symptoms among older people in Ethiopia. Therefore, this study aims to determine the prevalence of depressive symptoms and associated factors among the elderly population in the Gamo zone of southern Ethiopia.

**Methods:**

A community-based cross-sectional study was conducted among 840 randomly selected elderly individuals. A multi-stage sampling technique was employed to recruit participants. Depressive symptoms were assessed using the 15-item Geriatric Depression Scale (GDS) screening tool. Data collection was performed through face-to-face interviews. Descriptive statistics were initially computed. Subsequently, logistic regression analysis was conducted to identify independent factors associated with the outcome variable.

**Result:**

The finding of the study showed that the prevalence of depressive symptoms among older people living in the Gamo zone was 424(50.48%) (95% CI=47.09-53.86). Age 70–74 years [AOR=2.81, 95% CI 1.64-4.81], 75 years and above [AOR=5.09, 95% CI 3.00-8.64], age 65–69 years [AOR=2.43, 95% CI 1.62-3.66]; being widowed [AOR=2.73, 95% CI 1.69-4.42], ever chewing khat [AOR=5.89, 95% CI 1.17-29.53], being poor economic status [AOR=9.35, 95% CI 3.58-24.45], being average economic status [AOR=5.36, 95% CI 2.15-13.37], having 1–2 stressful life events [AOR=5.13, 95% CI 3.35-7.86], having 3 and above stressful life events [AOR=11.02, 95% CI 6.59,18.41], living alone [AOR=2.65, 95% CI 1.43-4.93] and those who lived with children [AOR=3.16, 95% CI 1.70-5.88] were significantly associated with depression.

**Conclusion:**

Half of the study participants exhibited depressive symptoms. Urgent interventions are essential to enhance psychological well-being and mitigate the impact of various modifiable risk factors associated with depression symptoms in elderly individuals. This includes increasing social support, particularly for those who have experienced stressful life events, live alone, or have low economic status. Healthcare providers should implement routine screening for depressive symptoms and offer supportive counseling. Policymakers and stakeholders should prioritize improving access to mental health services.

## Introduction

Globally, the number of individuals aged 60 and above is increasing at a rapid pace. In 2022, this demographic totaled 1.4 billion (1). By 2050, it is projected that 80% of all older people will reside in low and middle-income countries (2, 3). In Ethiopia, 4.7% of the total population falls within the elderly category (aged ≥ 60 years) (4). Elderly populations face distinct physical and mental health challenges that require recognition (1). This stage of life often coincides with emotional vulnerability and susceptibility to various diseases and recurring accidents (5). Apart from neurobiological changes in the brain, aging entails gradual, significant losses not only in emotional and physical well-being but also in social status over the years (1, 6). Common health conditions in older adults include hearing loss, cataracts, refractive errors, back and neck pain, osteoarthritis, diabetes, and depression (7).

Depression is a major public health concern worldwide, with significant implications for quality of life and functional capacity, particularly among older adults (8, 9). It is contributing significantly to the global burden of disability, with an estimated 264 million people affected worldwide (10). In Sub-Saharan Africa (SSA), depression is a prominent public health concern, with prevalence rates reaching 9% among the general population (11). Evidence indicates a rising prevalence of mental health issues among adults overall, particularly among the elderly (2). According to the World Health Organization (WHO), over 20% of individuals aged 60 years and older experience a mental health disorder, contributing to 6.6% of all disabilities in this age group (1). Depression is the most common mental health disorder among older adults, affecting approximately 5% of the global elderly population (1).

To enhance the health status of the elderly population, numerous initiatives have been undertaken at both national and international levels. For instance, Sustainable Development Goal 3 aims to ensure healthy lives and promote well-being for all ages, with Target 3.4 specifically focused on improving mental health and well-being by 2030 (12). In Ethiopia, mental health services have been integrated into the primary healthcare system in recent years (13). The country’s primary healthcare framework, which serves as the first level in its three-tier health service structure, includes primary hospitals, health centers, and satellite health posts (13). Health posts act as initial points of contact for patients, providing preventive and basic health services through home visits and at the health post itself. Health extension play a crucial role, delivering a comprehensive package of eighteen health services designed to prevent disease and promote better health outcomes. This entire primary healthcare system is overseen by district health offices to ensure effective delivery and monitoring (14, 15).

In Ethiopia, as in many other low- and middle-income countries, mental health issues among the elderly are increasingly recognized as a critical public health concern (1–4). Older adults in these settings face distinctive challenges that can exacerbate mental health problems, including declining physical health, diminished social support, and heightened exposure to various life stressors such as economic hardship and social isolation (5–7). Despite the growing awareness of these issues, there remains a significant gap in comprehensive data regarding the prevalence and determinants of depression among older adults in Ethiopia. This lack of detailed information hampers the development of targeted interventions and effective policies to address the mental health needs of this vulnerable population. Therefore, this study aims to fill this gap by assessing the prevalence of depression symptoms and identifying the associated factors among the elderly population in the Gamo Zone, Southern Ethiopia. The findings will provide valuable insights into the mental health challenges faced by older adults in this region and support the development of tailored strategies for prevention and intervention.

## Materials and methods

### Study setting and design

This study was conducted in the Gamo zone of South Ethiopia Regional State. Arba Minch town is the administrative center of this Zone. This town is 431 km from the Ethiopian capital (Addis Ababa). Ethiopia is administratively divided into four levels: regions, zones, *woredas* (districts), and *kebele* (wards). The first administrative division in Ethiopia is a *region. The second* administrative division is zones. Regions are subdivided into *zones*. The third administrative division *is woredas. A woreda is an administrative unit corresponding to districts in other parts of the world.* Zones are divided into *woredas* (districts). In addition to woredas, city administrations are considered at the same level as the woredas. *Woredas* (districts) are divided into *kebele*. This is the smallest administrative division. It is a collection of 3,000 to 5,000 inhabitants and the primary contact for most citizens living in Ethiopia. In the Gamo zone, six city administrations and 14 rural *woredas* (districts) with 325 *kebeles* were found. There are 302 health posts, 56 health centers, and six hospitals providing health services to the population.

### Population

All households in the selected study area served as the source population for this study. The study population included all randomly selected households in the area during the data collection period that met the inclusion criteria.

### Inclusion and exclusion criteria

The inclusion criteria required households to have elderly individuals (≥60 years old) who were permanent residents of the area for at least six months. However, individuals with severe depression, other severe illnesses, or hearing problems were excluded from participation.

### Sample size and sampling technique

The sample size was determined using OpenEpi Version 3.01 by following single population proportion assumptions of the expected prevalence of depression in the elderly population of 54.5% taken from a study conducted in North Shoa Area, Oromia Region, Ethiopia (6), 95% confidence level, 80% power, and 5% degree of precision. The sample size calculated using these assumptions was 382. With additional consideration of a design effect of two (for use of multi-stage sampling) and a 10% potential non-response rate, the final sample size was estimated to be 840. A multi-stage sampling technique was used to select study participants. Initially, six districts were selected from the total of 24 districts found in the Gamo zone using a lottery method. The selected districts were Bonke (with 10 *kebeles*), Kemba Zuria (with 27 *kebeles*), Boreda (with 29 *kebeles*), Gacho Baba (with 14 *kebeles*), Bibrbir town administrative (with 4*kebeles*) and Gerese town administrative (with 6 *kebeles*).

In the randomly selected districts, there are 90 *kebeles*, the lowest administrative units in Ethiopia, and 30% of these *kebeles* (27 *kebeles*) were included in the study. The sample size was proportionally allocated across each of the selected *kebeles*. With the assistance of health extension workers, sampling frames were created for each selected kebele using the master family index found in each kebele. Households with eligible elders were then selected using a simple random sampling technique. Finally, elders (≥ 60 years old) who were present in the household at the time of the visit and agreed to participate were interviewed. If there was more than one eligible participant in a selected household, one was chosen by the lottery method. For homes that were closed during our initial visit, we conducted up to three additional visits at different times of the day and on various days to maximize the likelihood of reaching potential participants. If the home remained closed after these attempts, we replaced it with the next household on our random selection list to maintain the sample size and ensure randomness.

### Operational definitions of terms

Aging Population: The definition of old age depends on various contexts of countries’ settings to determine the elder age cut point. The United Nations uses 60 years to define old age and recommends the age range of 50 to 65 years to be used as a cutoff point by countries (16). In Ethiopia, the cut points of elder age were started in 60 years (17, 18).

### Depressive symptoms

Depressive symptoms were assessed using the 15-item Geriatric Depression Scale (GDS) screening tool, where individuals self-reported their feelings over the past two weeks by answering questions with “Yes” or “No.” Each response indicating depression counted as one point, while the other response counted as zero. Scores on the scale range from 0 to 15. Based on the scores, 0–4 is considered normal, 5–8 indicates mild depressive symptoms, 9–11 signifies moderate depressive symptoms, and 12–15 indicates severe depressive symptoms (19). In this study, mild, moderate, and severe depressive symptoms were combined into a single depression symptoms category, reflecting the high likelihood of depression among the elderly experiencing stressors or problems. Thus, participants were categorized into two groups: those with depression symptoms and those without (20). The GDS has been validated in Africa and Ethiopia among older adults, showing satisfactory reliability with a Cronbach’s alpha of 0.81 and test-retest reliability of 0.73. The GDS was validated in Africa as well as in Ethiopia among older adults and was satisfactory with (Cronbach α=0.81) and test-retest reliability (r=0.73) (6, 7). In this study, the reported Cronbach’s alpha of the whole scale was 0.81.

#### Presence of stressful life events

Presence of stressful life events: refers to Individuals who had at least one or more stressful life events (close family member died, divorce, serious illness or injury in the family member, etc.….) in the last four weeks. The List of Threatening Experiences (LTE) developed by Brown & Harris (1978) was used to measure life events. The LTE contains 12 categories of significant life events, for example relating to the death of close persons, loss of relationships, imprisonment, and being the victim of theft. These 12 categories accounted for two-thirds of all events collected in the original development of the tool. Thus the experience of life events was grouped into three categories (none; 1–2 life events and 3 and above) (21). In this study, the reported Cronbach’s alpha of the whole scale was 0.84.

#### Perceived social support

Perceived social support: Social support has been described as support access to an individual through social ties to other individuals, groups, and the larger community (5). To assess perceived social support we will use Oslo’s 3-item social support scale. The OSSS-3 sum score can be operationalized into three broad categories of social support with a score of 3-8 indicating poor support, 9-11 moderate support, and 12-14 strong support (5, 22). In this study, the reported Cronbach’s alpha of the whole scale was 0.75.

### Data collection tools and procedure

Data collection was carried out using pre-tested and structured questionnaires. The data collection instrument was developed after reviewing previous literature (5, 6, 23, 24). The questionnaire had five parts; the first part was assessing socio-demographic-related information. The second part of the study focused on assessing depressive symptoms among the elderly population using the 15-item Geriatric Depression Scale (GDS) screening tool (6). This instrument has been adapted and used previously in the Ethiopian setting (3, 25).

The third part was assessing lifestyle-related information like current substance use. The fourth part was assessing comorbid chronic disease-related information. The last part was assessing psychosocial relationships like social support, and experience of life-threatening events. To assess the current substance use the participants were asked where they used at least one of the specified substances (alcohol, chewing khat, and tobacco) in the past 30 days (6). To assess perceived social support we used the Oslo 3-item social support scale (26).

The tool was initially developed in English, then translated into Amharic, and back-translated to English to ensure consistency. Nine trained health professionals with experience in data collection were selected as data collectors, and they were closely supervised by three supervisors with extensive data collection experience. The selection criteria for data collectors and supervisors included their ability to communicate in the local language and their prior data collection experience.

The principal investigator provided extensive two-day training for the supervisors and data collectors, focusing on questionnaire administration, maintaining confidentiality and privacy, and ensuring neutrality during interviews. Data were collected through face-to-face interviews during house-to-house visits to randomly selected homes, with interviews conducted in locations convenient for each respondent. Supervisors monitored the entire data collection process and checked the data for completeness daily. To increase the response rate, data collectors revisited participants who were not available during the initial visit at least three times.

### Data quality assurance

Data quality was ensured during instrument development, collection, coding, entry, and analysis. The data collectors and supervisors were trained about the purpose of the study and how to supervise and collect interviewer-administered questionnaires respectively. We pretested the questionnaire on 5% of the total sample size among elders in the Arba Minch Zuriya district, Gamo Zone, Southern Ethiopia. This pretesting was conducted outside of the study area to ensure the tool’s validity and reliability. This was not involved in the actual data collection. During data collection, the questionnaire was checked for its completeness daily by supervisors and then by investigators. If there was a problem encountered during data collection, there was a discussion with data collectors and supervisors accordingly. Intensive supervision was carried out by the assigned trained supervisors and research team members throughout the data collection period. This was help to identify problems that were addressed in the questionnaires.

### Data processing and analysis

Each questionnaire was checked for completeness entered into Epi-data version 3.1software and then exported to STATA version 14.0 for analysis. After data cleaning and editing, descriptive analysis was performed. The reliability of the measurements was assessed using Cronbach’s alpha for each composite variable. A Cronbach’s alpha score above 0.70 indicates a high level of internal consistency. The dependent variable in this analysis was depression symptoms (coded as 1 for those with depression symptoms and 0 otherwise). To identify factors associated with depression symptoms, a binary logistic regression analysis was performed for each independent variable. Variables with a P value ≤ 0.2 in the binary analysis were included in a multivariable logistic regression analysis to control for potential confounding effects (27). Additionally, contextually important and previously identified variables were included in the multivariable analysis.

The crude and adjusted odds ratios together with their corresponding 95% confidence intervals were computed and interpreted accordingly. To decide the level of significance, the p-value associated with each parameter was estimated using the p-value less than 0.05 as a cut point. Multi-collinearity assumptions were assessed using the variance inflation factor (VIF). The goodness-of-fit test for the final model was checked by Hosmer and Lemeshow’s goodness-of-fit model

### Ethics approval and consent to participate

Ethical approval for this study was obtained from the Institutional Review Committee of Arba Minch Health Sciences College on June 6, 2022 (project reference number: AMHSC/01/20/1100). Prior to the fieldwork, we communicated the overall purpose of the study to the relevant authorities and obtained written permission from the Gamo Zone Health Department. Verbal consent was also secured from village heads/mayors before conducting community surveys. Each participant was fully informed about the study’s purpose, benefits, potential risks, their anonymity, and their right to withdraw at any stage. Informed consent was obtained from each participant through a signature or thumbprint. Participation was entirely voluntary, and participants had the opportunity to ask questions and decline or cancel the interview at any point. We assured participants of the privacy and confidentiality of their information, emphasizing that the data collected would only be used for research purposes and not disclosed outside the research team. All methods adhered to the Helsinki Declaration guidelines.

## Results

### Socio-demographic characteristics of participants

A total of 840 participants took part in this study. More than half of 519 (63%) were male. The (mean ± SD) age of the participants was (68.7 ± 8.1) years. More than half, 648 (77.1%) were married and about 469 (55.8%) lived with their children while 86 (10.2%) lived with their spouse. More than half, 538 (64.0%) of the participants had no formal education. More than half of the respondents 594 (70.7%) live in the rural part of the zone and 559 (66.5%) had an average economic status compared to other households in the community. In terms of social support, almost half of 412 (49.0%) participants had poor social support. More than a quarter (27.3%) of the older population had three or more stressful life events and about 359 (42.7%) had one or two stressful life events (**Table 1**).

**Table 1:** Socio-demographic characteristics of the elderly population at Gamo Zone Southern Ethiopia 2024 (*n* = 840).

Variables	Categories	Frequency	Percentage
Age (years)	60-64	308	36.6
	65-69	251	29.9
	70-74	115	13.7
	≥75	166	19.8
Sex	Male	529	63.0
	Female	311	37.0
Marital status	Single	2	0.2
	Married	648	77.1
	Divorced	18	2.1
	Widowed	172	20.5
Residence	Urban	246	29.3
	Rural	594	70.7
Educational status	No formal education	538	64.0
	Primary(1-8)	240	28.6
	Secondary	30	3.6
	College and above	32	3.8
Economic status	Poor	214	25.5
	Average	560	66.7
	Wealthy	66	7.9
Living arrangements	Living with spouse	86	10.2
	Living alone	284	33.9
	Living with children	470	55.8
Ever chewing chat	Yes	14	1.7
	No	826	98.3
Perceived social support	Poor social support	412	49.0
	Moderate Social Support	352	41.9
	Strong Social Support	76	9.0
Stressful life event	None	252	30.0
	1-2	359	42.7
	3 and above	229	27.3

### Prevalence of depression among the elderly population

The result of the study showed that the prevalence of depression among older people in the Gamo zone was 424 (50.5%) (95% CI = 47.1-53.9). Regarding the levels of depression symptoms, the majority of individuals (315, or 37.5%) have mild depression symptoms, while 94 (11.2%) have moderate symptoms, and 15 (1.8%) have severe symptoms. However, 416 respondents (49.5%) do not have any depression symptoms. About 634 (75.5%) of the older population were generally dissatisfied with their lives. More than three-quarters (76.6%) of the older population preferred to stay at home rather than go out and do new things. Almost a third (32.4%) of the participants often felt bored. About 348 (41.4%) of the elderly population often felt helpless (**Table 2**).

**Table 2:** Prevalence of Geriatric Depression among the elderly population at Gamo Zone Southern Ethiopia 2024 (*n* = 840).

Variables	Categories N (%)	
	Yes	No
Are you satisfied with your life?	206(24.5)	634(75.5)
Have you dropped many of your activities or interests?	386(45.9)	454(54.1)
Do you feel that your life is empty?	187(22.3)	653(77.7)
Do you often feel bored?	272(32.4)	568(67.6)
Are you in good spirits most of the time?	523(62.3)	317(37.7)
Are you afraid that something bad is going to happen to you?	276(32.9)	564(67.1)
Do you feel happy most of the time?	528(62.9)	312(37.1)
Do you often feel helpless?	348(41.4)	492(58.6)
Do you prefer to stay at home, rather than go out and do new things?	643(76.6)	197(23.4)
Do you feel you have more problems with your memory than most?	197(23.4)	643(76.6)
Do you think it is wonderful to be alive?	754(89.8)	86(10.2)
Do you feel pretty worthless the way you are now?	551(65.6)	289(34.4)
Do you feel full of energy?	398(47.4)	442(52.6)
Do you feel that your situation is hopeless?	136(16.2)	704(83.8)
Do you think that most people are better off than you are?	436(51.9)	404(48.1)

### Factors associated with depression among the elderly population

The finding of this study reveals that variables like age, living arrangement, marital status, economic status, stressful life events, and substance use were significantly associated with depression during multivariable analysis. However, depression had no significant association with social support, educational status, and place of residents. Those respondents whose age was between 65-69 years and those aged above 75 years were 2.4 (AOR = 2.4, 95% CI 1.6, 3.7) and 5.1 (AOR = 5.1, 95% CI 3.0, 8.6) times more likely to develop depression than those whose age was between 60-64 years respectively. The odds of developing depression were 2.7 (AOR = 2.7, 95% CI 1.6 -4.4) times higher among widowed individuals than married individuals. The odds of developing depression were 9.4 (AOR=9.4, 95% CI, *3.6, 24.5* ***)*** times higher among those whose economic status was poor compared to those who had good economic status. Those elders who lived alone and those who lived with children were 2.7 (AOR=2.7, 95% CI 1.4, 4.9) and 3.2 (AOR=3.2, 95%CI 1.7, 5.9) times more likely to develop depression than those who lived with their spouse respectively. Elders who had 1–2 stressful life events and those who had 3 and above stressful life were 5.1 (AOR=5.195%CI 3.4, 7.9) and 11.0 (AOR=11.0,95% CI 6.6, 18.4) times more likely to develop depression than those who hadn’t stressful life events respectively. The odds of developing depression were 5.9 (AOR=5.9 (1.2, 29.5) times higher among khat chewers than those who did not chew khat (**Table 3**).

**Table 3:** Factors associated with depression among the elderly population in Gamo Zone, Southern Ethiopia, 2024.

Variables	Categories	Depression		COR (95%CI)	AOR (95%CI)	P-value
		Yes	No			
**Age years**	60-64	101 (32.8)	207 (67.2)	1	1	1
	65-69	130 (51.8)	121 (48.2)	2.2 (1.6,3.1)	***2.4 (1.62,3.66)***	0.001**
	70-74	74 (64.4)	41 (35.6)	3.8 (2.4,5.9)	***2.8 (1.64,4.81)***	0.001**
	75 and Above	119 (71.7)	47 (28.3)	5.3 (3.5,7.8)	***5.1 (3.00,8.64)***	0.001**
**Sex**	Male	250 (47.3)	279 (52.7)	1	1	1
	Female	174 (55.9)	137 (44.1)	1.4 (1.1,1.9)	1.3 (0.89,1.84)	0.191
**Marital status**	Married	283 (44.1)	362 (55.9)	1	1	1
	Divorced	16 (80.0)	4 (20.0)	5.1 (1.7,15.3)	3 (0.8, 10.8)	0.092
	Widowed	122 (70.9)	50 (29.1)	3.1 (2.2,4.4)	***2.7 (1.7, 4.4)***	0.001**
**Place of residence**	Urban	127 (51.6)	119 (48.4)	1.1 (0.8,1.4)	1.2 (0., 1.8)	0.474
	Rural	297 (50.0)	297 (50.0)	1	1	1
**economic status**	Poor	144 (67.3)	70 (32.7)	17.3 (7.5,39.9)	***9.4 (3.6, 24.5)***	0.001**
	Average	273 (48.8)	287 (51.2)	8.0 (3.7,17.9)	***5.4 (2.2,13.4)***	0.001**
	Wealthy	7 (10.6)	59 (89.4)	1	1	1
**Educational status**	No formal education	295 (54.8)	243 (45.2)	2.7 (1.245.8)	1.2 (0.5,3.2)	0.684
	Primary (1-8)	105 (44.1)	133 (55.9)	1.7 (0.8,3.8)	1.2 (0.5, 3.2)	0.722
	Secondary (9-12)	13 (43.3)	17 (56.7)	1.7 (0.7,4.8)	1.8 (0.5,6.5)	0.377
	College and above	10 (31.3)	22 (68.8)	1	1	1
**Living arrangements**	Living with spouse	38 (44.2)	48 (55.8)	1	1	1
	Living alone	145 (51.1)	139 (48.9)	1.3 (0.8,2.1)	***2.7 (1.4,4.9)***	0.002*
	Living with children	241 (51.3)	229 (48.7)	1.3 (0.8,2.1)	***3.2 (1.7,5.9)***	0.001**
**Social support**	Poor social support	238 (57.8)	174 (42.2)	3.4 (1.9,5.7)	1.7 (0.9,3.3)	0.104
	Moderate Social Support	164 (46.6)	188 (53.4)	2.1 (1.3,3.7)	1.3 (0.7,2.4)	0.471
	Strong Social Support	22 (28.9)	34 (71.1)	1	1	1
**Stressful life event**	None	60 (23.8)	192 (76.2)	1	1	1
	1-2	206 (57.4)	153 (42.6)	4.3 (3.0,6.2)	***5.1 (3.4,7.9)***	0.001**
	3 and above	158 (69.0)	71 (31.0)	7.1 (4.8,10.7)	***11.0 (6.6,18.4)***	0.001**
**Used tobacco**	Yes	64 (60.4)	42 (39.6)	1.6 (1.12.5)	1.5 (0.9,2.5)	0.105
	No	360 (49.0)	374 (51.0)	1	1	1
**Chew khat**	Yes	11 (78.6)	3 (21.4)	3.7 (1.0,13.2)	***5.8 (1.2,29.5)***	0.031*
	No	413 (50.0)	413 (50.0)	1	1	1

**p-value < < 0.05, *1 references group*.

The bold and italicized values indicate statistically significant results.

## Discussion

This study aimed to assess the prevalence of depression symptoms and identify associated factors among the elderly population in the Gamo zone of southern Ethiopia. The findings reveal that 50% of the elderly individuals in this area exhibit symptoms of depression. Key socio-demographic factors linked to depression include age, marital status, and living environment. Additionally, the study underscores that economic vulnerability, as indicated by wealth status, along with exposure to stressful life events, is associated with an increased risk of depression symptoms among the elderly in the region.

The study revealed that the prevalence of depression among elderly individuals in the Gamo zone was 50.5% (n = 424; 95% CI = 47.1-53.9). This prevalence is comparable to rates observed in studies from India (52.4%) (28), China (52.9%) (29), North Shoa Zone (54.5%) (6), and North West Ethiopia (45.9%) (23). However, it is lower than prevalence reported in a community-based study conducted among the elderly in the Debre Libanos District, North Shoa Zone, Oromia Regional State, Ethiopia(59.9%) (17). Conversely, the prevalence in this study was notably higher compared to findings from Nepal (15.4%) (30) and Western India (16.75%) (31). The differences could lie in the relatively smaller sample size in the above study, which was expected to increase the probability of standard error, resulting in a less precise and reliable result with lower power compared to the current study. The differences could also be related to cultural differences, as the study was conducted in rural communities in Nepal, where social support networks may have a protective function and contribute to the low prevalence of depression. Additionally, measures of depression, typically the Major Depression Inventory Scale (MDI scale), have been criticized for their use in older people for being too long with a six-point Likert, meaning their use in an Indian study reported could explain low depression rates.

In this study, age was found to be significantly associated with depression. Older age groups, particularly those 65 years and older, exhibited a higher likelihood of depression compared to younger elderly individuals. This finding is consistent with research conducted in various regions, including Southern Ethiopia North-West Ethiopia, the North Shoa Zone of Oromia Regional State, North Shoa Zone (6). (5, 17, 23, 32), India (28), China (29) and Iran (33). The higher prevalence of depression among older adults is likely due to declines in physical function, increased vulnerability to chronic diseases, and greater exposure to negative emotions. These factors collectively contribute to the higher rates of depressive symptoms observed in older age groups. This underscores the need for targeted interventions to address mental health challenges in the elderly. Healthcare providers and policymakers should implement age-specific strategies, such as improving access to mental health services, promoting physical health programs, and establishing community support networks. These measures could reduce depression rates and enhance the quality of life for older individuals.

In this study, marital status was significantly associated with depression symptoms. Widowed older adults were notably more likely to experience depression symptoms compared to their married counterparts. This finding aligns with previous research identifying widowhood as a risk factor for depression symptoms among older adults in North West Ethiopia and India (5, 23, 34). The increased likelihood of depression symptoms among widowed individuals may be attributed to the negative emotions and feelings of emptiness following the loss of a spouse, which can lead to social withdrawal and depressive symptoms. The findings highlight the need for targeted support for widowed elderly individuals. Healthcare providers and policymakers should develop specialized programs to address the unique emotional and social challenges faced by this group. Effective interventions might include counseling services, social engagement activities, and community support groups designed to reduce isolation and enhance mental well-being.

The study also found a significant positive association between living arrangement and depression symptoms. Older adults who lived alone or with children were at a higher risk of depression symptoms compared to those living with a spouse. This finding is consistent with studies conducted in Ambo, Ethiopia (24), North Shoa Zone (6); Nigeria (7); and Myanmar (35). The higher risk of depression among those living alone or with children may be attributed to feelings of loneliness and unmet social needs, which can contribute to depressive symptoms. Additionally, a lack of independence, whether physical or financial, might also exacerbate depression among older adults (24). These findings highlight the need for tailored support interventions for older individuals, especially those living alone or with children. Healthcare providers and policymakers should develop programs to address social isolation and enhance social engagement. Community initiatives, such as support groups and social activities, could help alleviate loneliness and improve the mental well-being of elderly individuals in these living arrangements.

In addition, the study also found an inverse association between economic status and depression symptoms among older adults. Those with poor or average economic status were at a higher risk of depression symptoms compared to their wealthier counterparts. This finding is consistent with research from China (29); Nepal (30); and Ghana (10), which suggests that higher income acts as a protective factor against depression in older adults. The increased likelihood of depression among socioeconomically disadvantaged individuals may be attributed to greater psychological distress arising from limited household resources and financial stress. These findings highlight the need for economic support and financial assistance programs for older adults. Policymakers should consider implementing measures to improve the economic security of disadvantaged elderly individuals, such as targeted financial aid, social welfare programs, and access to resources that can alleviate financial stress. Enhancing economic stability may help reduce the risk of depression and improve overall mental well-being in this population.

Moreover, the study found that stressful life events are significant risk factors for depression symptoms among older adults. Individuals who experienced stressful events were more likely to suffer from depression compared to those without such experiences. This finding is consistent with research from Italy (36) and Nepal (30), which also found a link between stressful events and depression. Stressful life events can impair an individual’s ability to cope with adversity, contributing to an increased risk of depressive symptoms. These findings suggest the importance of providing psychological support and stress management resources for older adults. Healthcare providers and policymakers should consider developing interventions that help older individuals manage stress and build resilience. This might include access to counseling services, stress reduction programs, and community support initiatives aimed at mitigating the impact of stressful life events on mental health.

Furthermore, the study found an association between substance use, specifically khat chewing, and depression symptoms. Older adults who chewed khat were more likely to experience depression symptoms compared to those who did not. This finding contrasts with study from the Womberma District in northwestern Ethiopia, which found no association between khat use and depression in the elderly (5). The discrepancy may be due to the withdrawal effects of khat or other factors influencing mental health in those currently using the substance. These findings highlight the need for targeted interventions addressing substance use among older adults. Healthcare providers should consider incorporating substance use screening into routine assessments and offer support for those struggling with substance-related issues. Additionally, public health initiatives should focus on raising awareness about the mental health risks associated with khat use and provide resources for cessation support to mitigate its impact on depression.

### Limitation of study

One limitation is the exclusion of older individuals with severe illnesses, severe depression, or hearing loss, which may restrict the generalizability of the findings to the broader elderly population. Additionally, as a cross-sectional study, it is challenging to establish causal relationships between variables and outcomes, highlighting the need for longitudinal studies to explore causality more effectively. The classification of khat use based on lifetime use does not capture the quantity, frequency, or duration of consumption, and future research should include detailed measures to assess these aspects. Moreover, important variables such as occupational status, family history of mental illness, comorbid physical illnesses, physical impairment, sleep disturbance, and alcohol use were not included in the analysis; incorporating these factors in future studies could provide a more comprehensive understanding of depression. Lastly, the findings may be influenced by cultural and social norms specific to the study area, which might not be applicable to other regions or populations.

### Conclusion and recommendations

The prevalence of depression among elderly people in the Gamo zone was high (50.5%). As in this study, depression was found to be associated with older age, widowhood, living with children or alone, poor economic status, exposure to stressful life events, and substance use. However, the high burden of depression among older persons who were exposed to vulnerabilities in this community can be considered as an early warning and calls for ongoing and additional investigation to quantify the severity of this impact through estimates derived via diagnostic instruments alongside widely used screening questionnaires According to the results of this study, recommended mitigation strategies should incorporate ways to promote mental wellbeing and target determinants of poor mental health, as well as interventions to treat those who develop a mental disorder where: Policy-makers need to issue policies that alleviate elderly persons’ socioeconomic disadvantages such as retirement from work. Healthcare workers should routinely screen elderly patients, particularly those with chronic conditions, in the healthcare system and the community for depression. Community leaders and members should be encouraged to build social support networks that improve the well-being of older people and thus build their resilience to stressful life events. Family members and relatives must be informed about the nature of depression so that they can identify affected elders early and provide support in preventing the onset of symptoms associated with the perceived loneliness sensation. Potential elderly people must prioritize avoiding unhealthy lifestyles and habits such as chat chewing. Prospective researchers planning to study depression in the elderly must consider diagnostic measures in addition to widely used screening questionnaires designed specifically for use in the elderly.

## Data Availability

The raw data supporting the conclusions of this article will be made available by the authors, without undue reservation.
